# Problem based learning.…Need for the hour

**DOI:** 10.4103/0972-0707.44043

**Published:** 2008

**Authors:** A Parameswaran

**Affiliations:** Former Principal, Tamilnadu Govt. Dental College, Former Prof & Head, Meenakshi Ammal Dental College, Chennai, India

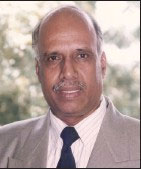



Education is seen by the humankind as an indispensable asset for excelling, today. Education should face the vision of the ‘new world order’ to enable the learner to access information and utilize any facility to its maximum potential.

Several educational innovations have been made, tried and have progressed across the world to create a change. Teaching and learning has progressed from being passive and didactic to being active and energetic. Among the innovations, Problem Based Learning (PBL) has made the greatest impact in providing adequate training in real-life management skills for the learners.

In the Indian scenario, PBL needs to be put on trial aggressively, because it –

- Encourages an open ended, reflective, active and critical learning experience.
- Offers the respect due to the teacher and the learner as persons with knowledge, understanding, and feelings.
- Excites and creates interest in all those who come together in a shared educational process.
- Reflects the true nature of the knowledge of the society.

Problem based learning motivates to learn professionally relevant material by encouraging a search beyond one's preconceptions. It is a novel learning strategy, which enables students to escape traditional lectures and textbooks that promote memorization. It enhances self-directed learning and decision making. The learner works on a problem with the ability to reason and apply his/her knowledge.

The part played by the teacher is to facilitate the students in identifying areas of learning appropriately. The teaching and learning of PBL give emphasis on “learner centered learning” and “problem centered learning”. The learning closely simulates the future practice environment and encourages students to adopt professional behavior and approach to patient care.

Many universities all over the world have changed the traditional style of curriculum which is predominantly “Lecture Based”. Students accept PBL, as it encourages thinking and active integration of information which ultimately helps in improving their skills. However, to have a change in curriculum needs a *mind set among the teachers* to accept, as the amount of content is overwhelming and much of the material that is taught seems remote.

The tutors are trained to facilitate the discussion mechanism. They should be able to explain and guide a group of students, practice by debating the issues and work as a team. The tutors should use “open-ended questions” effectively to facilitate discussion. They should provide feedback on the performance to the group.

Two-day workshops can be conducted for the facilitators to train them on PBL Process, Facilitation Process and Student assessment and Feedback.

The PBL method of teaching will open better opportunities for learners to develop more confidence, maturity and strength to deal with various clinical situations. In courses like Dentistry, Architecture, and Engineering, PBL should additionally include ‘Hands-on training’ to develop the psychomotor skill of the learner and make them better *doers* as well as *thinkers*.

Modern facilities and equipment have eased the lifestyle of people in many ways. The use of computers added a new dimension to the working style of many. Basic knowledge of computer operations seems to be essential, as it will aid in acquiring vast information faster. The thoughts can be viewed directly in print and can be transmitted immediately through the on-line facility of the journal.

I wish to share some thoughts with you, Editor of JCD. The accessibility of the on-line journal will be so useful in E-learning. I wish to congratulate you and your teams for making a beginning in assisting the students of conservative dentistry develop their knowledge and skill.

